# Hepatic Arterial Infusion Chemotherapy Combined With PD-1 Inhibitors Plus Lenvatinib *Versus* PD-1 Inhibitors Plus Lenvatinib for Advanced Hepatocellular Carcinoma

**DOI:** 10.3389/fonc.2021.618206

**Published:** 2021-02-25

**Authors:** Jie Mei, Yu-Hao Tang, Wei Wei, Ming Shi, Lie Zheng, Shao-Hua Li, Rong-Ping Guo

**Affiliations:** ^1^ Department of Liver Surgery, Sun Yat-Sen University Cancer Center, Guangzhou, China; ^2^ State Key Laboratory of Oncology in South China, Guangzhou, China; ^3^ Collaborative Innovation Center for Cancer Medicine, Guangzhou, China; ^4^ Department of Medical Imaging, Sun Yat-Sen University Cancer Center, Guangzhou, China

**Keywords:** hepatocellular carcinoma, hepatic artery infusion chemotherapy, programmed cell death protein-1, lenvatinib, FOLFOX

## Abstract

**Background:**

Lenvatinib combined with programmed cell death protein-1 (PD-1) inhibitors has resulted in good survival outcomes in the treatment of unresectable hepatocellular carcinoma (HCC). Hepatic artery infusion chemotherapy (HAIC) has also attracted attention due to its high response rates and favorable survival for advanced HCC patients. The present study aimed to compare the efficacy of HAIC combined with PD-1 inhibitors plus lenvatinib (HPL) and PD-1 inhibitors plus lenvatinib (PL) in patients with advanced HCC.

**Methods:**

Between July 2018 and December 2019, patients diagnosed with advanced HCC who initially received HPL or PL treatment were reviewed for eligibility. Efficacy was evaluated according to tumor response and survival.

**Results:**

In total, 70 patients met the criteria and were included in the present study, and they were divided into the HPL group (n = 45) and PL group (n = 25). The overall response rate (40.0 *vs*. 16.0%, respectively; p = 0.038) and disease control rate (77.6 *vs*. 44.0%, respectively; p < 0.001) were higher in the HPL group than in the PL group. The median overall survival was 15.9 months in the HPL group and 8.6 months in the PL group (p = 0.0015; HR = 0.6; 95% CI 0.43–0.83). The median progression-free survival was 8.8 months in the HPL group and 5.4 months in the PL group (p = 0.0320; HR = 0.74; 95% CI 0.55–0.98).

**Conclusion:**

Compared to PL, HPL was associated with a significantly better treatment response and survival benefits for patients with advanced HCC.

## Introduction

Hepatocellular carcinoma (HCC) is one of the most common malignancies and the fourth leading cause of cancer-related deaths worldwide (1). For advanced HCC, surgical resection is inapplicable, and locoregional approaches bring little benefit (2). Lenvatinib and programmed cell death protein-1 (PD-1) inhibitors are currently well-studied and proven to bring survival benefit as first- and second-line treatment of advanced HCC (3–5). In an open-label multicenter study, lenvatinib plus pembrolizumab surprisingly showed a median overall survival (OS) of 22 months and a median progression-free survival (PFS) of 8.6 months in patients with unresectable hepatocellular carcinoma (6). In recent years, hepatic artery infusion chemotherapy (HAIC) has attracted attention due to high response rates and favorable survival for advanced HCC (7). Several randomized clinical trials have shown that HAIC combined with sorafenib yields significantly better survival compared to sorafenib monotherapy (8, 9). These findings imply that HAIC may have potential when combined with targeted drug therapy.

To date, no research has studied the efficacy of HAIC in combination with lenvatinib and PD-1 inhibitors. Therefore, we designed this retrospective study to compare the survival of patients with advanced HCC who received HAIC combined with lenvatinib plus PD-1 inhibitors (HPL) *versus* those who received lenvatinib plus PD-1 inhibitors (PL), aiming to provide a reference for the treatment of advanced HCC.

## Methods

This study was conducted according to the ethical guidelines of the 1975 Declaration of Helsinki. The analysis of the patient data was reviewed and approved by the Institutional Review Board and Human Ethics Committee at the Sun Yat-Sen University Cancer Center (SYSUCC; Guangzhou, China).

### Patients

Between July 2018 and December 2019, the medical records of patients diagnosed with HCC who received HPL and PL treatment at the Department of Liver Surgery of SYSUCC were reviewed for eligibility. Patients were included based on the following specific criteria: a) patients were diagnosed with HCC through imaging or pathology according to the AASLD practice guidelines (10); b) no cancer-related therapies were involved before or during HPL or PL; c) patients had a tumor classification of Barcelona Clínic Liver Cancer (BCLC) B or C; d) Child-Pugh (CP) was classified as A or B; e) patients had at least two cycles of HPL or PL; f) no other malignant tumors were diagnosed; and g) complete medical and follow-up data were available. All laboratory serum test data were collected within 3 days before the initial treatment. Imaging evaluation included enhanced computed tomography (CT) or magnetic resonance imaging (MRI) examination within a week before the initial treatment.

### Treatment Procedure

Lenvatinib (The UK, Eisai Europe Co. Ltd.) (8 to 12 mg according to bodyweight) was taken orally. PD-1 inhibitors were used intravenously at the standard dose (**Table S1**). The first use of PD-1 inhibitors was within 7 days of initiation of lenvatinib. For the HPL group, HAIC was administered according to previously described procedures (11). Femoral artery puncture and catheterization were performed in every cycle of treatment. The FOLFOX regimen was administered *via* the hepatic artery as follows: 85 or 135 mg/m^2^ oxaliplatin, 400 mg/m^2^ leucovorin, and 400 mg/m^2^ fluorouracil on the first day; and 2400 mg/m^2^ fluorouracil over 46 h. Patients received PD-1 inhibitors and lenvatinib within 3 days before or after the start of HAIC. The discontinuation of treatment depended on disease progression, unacceptable toxicity, patient withdrawal of consent, or changes of treatment plan. The final follow-up ended on September 30, 2020. Enhanced CT or MRI was performed every 2 or 3 months. Routine follow-up intervals were 2 to 4 months.

### Diagnosis and Definitions

Tumor response was defined as complete response (CR), partial response (PR), stable disease (SD), or progressive disease (PD) according to the modified Response Evaluation Criteria in Solid Tumors 1.1 (mRECIST) (12). Overall response rate (ORR) was calculated as the sum of CR and PR. Disease control rate (DCR) was calculated as the sum of CR, PR, and SD. Overall survival (OS) was defined as the time interval from treatment initiation to cancer-related death. Progression-free survival (PFS) was defined as the time interval from treatment initiation to progression or death. Treatment-related adverse events (AEs) were evaluated by National Cancer Institute Common Terminology Criteria for Adverse Events version 4.0.

### Statistical Analysis

Categorical variables in the baseline characteristics were compared using the Pearson’s χ2 test or Fisher’s exact test. Variable distribution was described using mean ± standard error (SE) for normally distributed values, and median and range were used for non-normally distributed values. Survival analysis was calculated using the Kaplan-Meier method, and differences in the survival curves were analyzed with a log-rank test. All variables with a P value < 0.05 in univariate analyses were used in multivariate analyses using Cox regression models. The hazard ratio (HR) and confidence intervals (CI) were calculated. A two-tailed P value < 0.05 was considered statistically significant. All data analyses were performed using SPSS 25.0 software (SPSS Inc., Chicago, IL) and GraphPad Prism (version 8.0; GraphPad, Inc.).

## Results

### Identification and Characteristics of Study Patients

**Figure 1:**
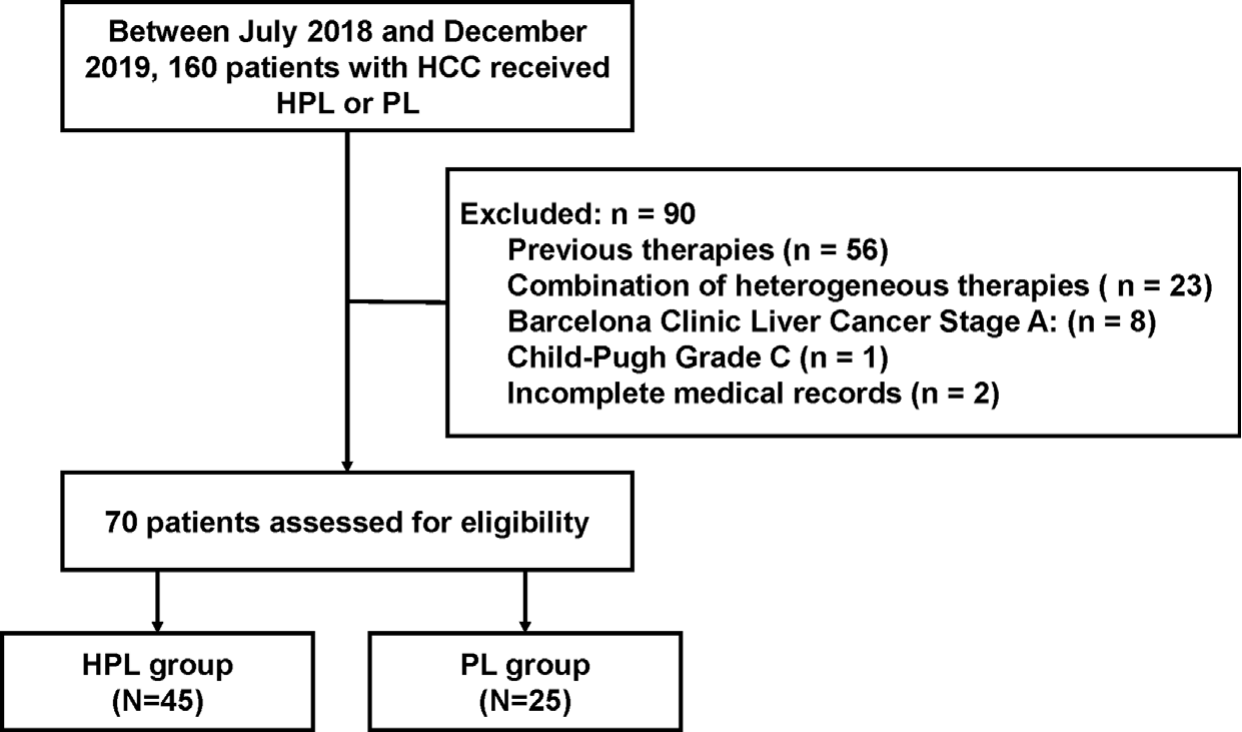
Flow diagram summarizing the disposition process of patients.

From July 2018 to December 2019, 160 patients with HCC who received HPL or PL were screened: 56 patients received previous surgery, interventional therapies, tyrosine kinase inhibitors or immune-targeted therapies; 23 patients participated in other treatments during HPL or PL; 8 patients were classified with a tumor grade of BCLC/A; 1 patient was classified as CP C; and 2 patients had missing sections in their medical records. Finally, a total of 70 patients who met the criteria were included in the study, and the patients were divided into the HPL group (n = 45) and PL group (n = 25). The patient characterization process is shown in **Figure 1**. Of note, the treatment of PD-1 inhibitors plus lenvatinib was available since July 2018 at our center.

The clinical characteristics and treatment of patients are summarized in **Table 1**. Most patients were classified into CP A (97.8% in the HPL group and 88% in the PL group) and BCLC/C (88.9% in the HPL group and 88.0% in the PL group). Two groups were comparable in the clinical characteristics, liver function, and tumor characteristics. A higher proportion of patients in the PL group had extrahepatic metastasis compared to the HPL group (52.0 *vs*. 33.3%), but the difference was not statistically significant (p = 0.127). In the HPL group, the cycles of PD-1 inhibitors plus lenvatinib ranged from 2 to 12, with a median of 5. While in the PL group, the cycles of PD-1 inhibitors plus lenvatinib ranged from 2 to 9, with a median of 4. The PD-1 inhibitor categories in each group are summarized in **Table S1**.

**Table 1:** Baseline clinical characteristics of patients.

Characteristic*	HPL (n=45)	PL (n=25)	P value
Age	49.1 ± 10.6	50.1 ± 12.3	0.366
Gender*			0.212
Male	38 (84.4)	18 (72.0)	
Female	7 (15.6)	7 (28.0)	
HBV			0.533
Negative	8 (17.8)	6 (24.0)	
Positive	37 (82.2)	19 (76.0)	
HCV			1.000
Negative	44 (97.8)	25 (100.0)	
Positive	1 (2.2)	0 (0)	
WBC (10E9/L)	6.7 (5.6, 8.0)	7.4 (5.7, 9.7)	0.420
NEU (10E9/L)	4.78 (3.66, 5.92)	4.86 (3.20, 7.20)	0.876
LYM (10E9/L)	1.2 (0.9, 1.6)	1.4 (1.1, 1.8)	0.210
HB (g/L)	138.4 ± 20.7	137.9 ± 29.6	0.944
PLT (10E9/L)	227.0 (184.0, 328.0)	201.0 (149.8, 281.5)	0.351
ALT (U/L)	45.5 (34.0, 74.4)	57.0 (37.6, 89.8)	0.329
AST (U/L)	76.2 (55.6, 152.1)	96.7 (56.7, 173.4)	0.655
AFP (ng/ml)	4106.0 (72.8, 121000.0)	767.6 (23.3, 21940.5)	0.193
PIVKA-II (mAU/ml)	9929.0 (1672.0, 51343.0)	11794.5 (252.3, 75000.0)	0.952
Liver cirrhosis			0.143
Absent	5 (11.1)	7 (28.0)	
Present	40 (88.9)	18 (72.0)	
Child-Pugh			0.127
A	44 (97.8)	22 (88.0)	
B	1 (2.2)	3 (12.0)	
Barcelona Clínic Liver Cancer			1.000
B	5 (11.1)	3 (12.0)	
C	40 (88.9)	22 (88.0)	
Size of largest nodule (cm)	11.2 ± 3.9	10.9 ± 4.2	0.754
Tumor number			1.000
Solitary	9 (20.0)	5 (20.0)	
Multiple	36 (80.0)	20 (80.0)	
Tumor distribution			0.409
Uni-lobar	17 (37.8)	7 (28.0)	
Bi-lobar	28 (62.2)	18 (72.0)	
Tumor thrombus			0.237
Absent	9 (20.0)	7 (28.0)	
Branch of portal vein	20 (44.4)	6 (24.0)	
Main portal vein	16 (35.6)	12 (48.0)	
Extrahepatic metastasis			0.127
Absent	30 (66.7)	12 (48.0)	
Present	15 (33.3)	13 (52.0)	

*No (%).

PD-1, programmed cell death protein-1; HPL, hepatic artery infusion chemotherapy combined with PD-1 inhibitors plus lenvatinb; PL, PD-1 inhibitors plus lenvatinib; HBV, hepatitis B virus; HCV, hepatitis C virus; WBC, white blood cell; NEU, neutrophil; LYM, lymphocyte; HB, haemoglobin; PLT, blood platelet; ALT, alanine aminotransferase; AST, aspartate aminotransferase; AFP, alpha fetoprotein; PIVKA-II, protein induced by vitamin K absence or antagonist-I.

### Survival

**Figure 2:**
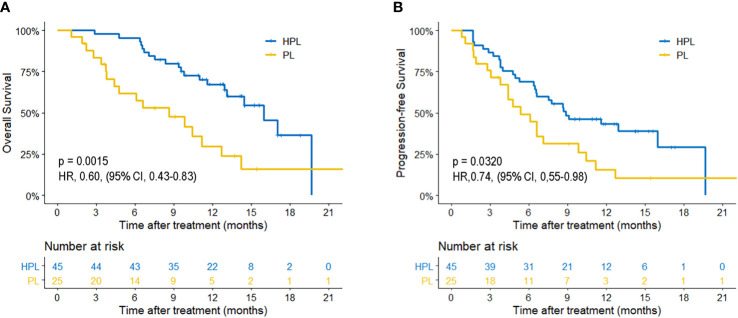
Kaplan-Meier curves of survival outcomes of patients in the two groups. **(A)** Overall survival. **(B)** Progression-free survival. HPL, hepatic artery infusion chemotherapy combined with PD-1 inhibitors plus lenvatinib; PL, PD-1 inhibitors plus lenvatinib.

**Figure 3:**
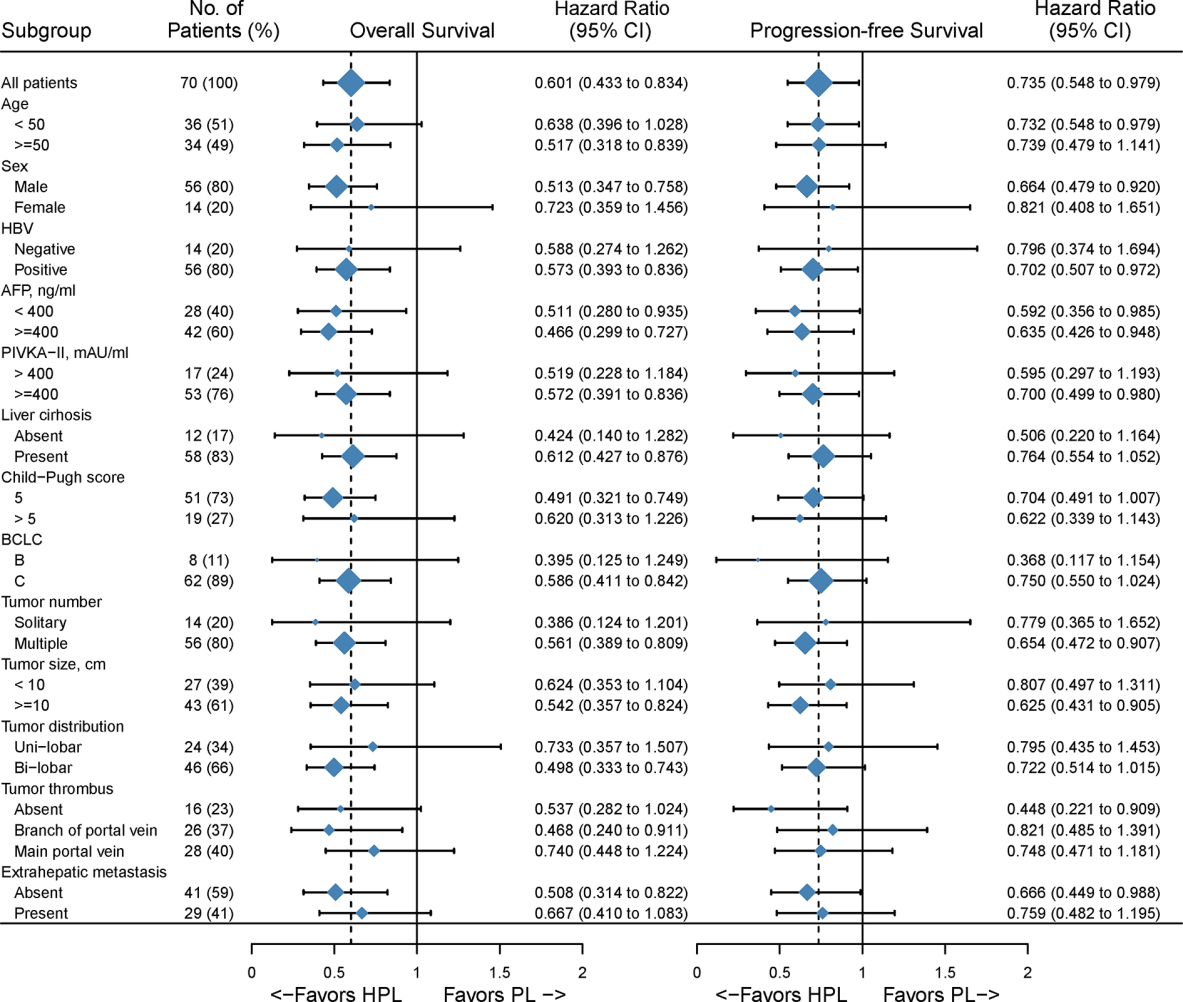
Forest plot for overall survival of the matched cohorts of patients. HPL, hepatic artery infusion chemotherapy combined with PD-1 inhibitors plus lenvatinib; PL, PD-1 inhibitors plus lenvatinib.

The median follow-up time was 15.1 months. Patients in the HPL group had significantly better survival outcomes than those in the PL group. The 3-, 6-, and 12-month OS was 97.8, 86.7, and 67.4%, respectively, in the HPL group, and 83.6, 61.8, and 29.8%, respectively, in the PL group. The median OS was 15.9 months in the HPL group and 8.6 months in the PL group (p = 0.0015; HR = 0.6; 95% CI 0.43–0.83). The 3-, 6-, and 12-PFS was 86.7, 68.9, and 43.2%, respectively, in the HPL group, and 75.8, 49.2, and 15.7%, respectively, in the PL group. The median PFS was 8.8 months in the HPL group and 5.4 months in the PL group (p = 0.0320; HR = 0.74; 95% CI 0.55–0.98). The survival curves are shown in **Figure 2**. The forest plot analysis of factors associated with OS and PFS is shown in **Figure 3**. HPL provided a clinical benefit in patients with large, multiple HCCs, but it failed to have a survival benefit in patients with main portal vein tumor thrombus or extrahepatic metastasis.

### Tumor Response

The treatment response is summarized in **Table 2**. Based on mRESIST, the ORR was higher in the HPL group (40.0%) than in the PL group (16.0%) (p = 0.038). A higher DCR in both overall response (77.6 *vs*. 44.0%; p < 0.001) and intrahepatic response (88.9 *vs*. 52.0%; p = 0.001) was present in the HPL group compared to the PL group.

**Table 2:** Summary of best response.

Variable	HPL (n=45) No. (%)^c^	PL (n=25) No. (%)^c^	P value
**Overall response****^a^**			
Complete response	0 (0.0)	0 (0.0)	1.000
Partial response	18 (40.0)	4 (16.0)	0.038
Stable response	20 (44.4)	7 (28.0)	0.176
Progressive response	5 (11.1)	6 (24.0)	0.156
Not assessable	2 (4.4)	8 (32.0)	0.002
Overall response rate	18 (40.0)	4 (16.0)	0.038
Disease control rate	38 (77.6)	11 (44.0)	<0.001
**Intrahepatic response****^b^**			
Complete response	0 (0.0)	0 (0.0)	1.000
Partial response	18 (40.0)	4 (16.0)	0.038
Stable response	22 (48.9)	9 (36.0)	0.298
Progressive response	3 (6.7)	4 (16.0)	0.212
Not assessable	2 (4.4)	8 (32.0)	0.002
Overall response rate	18 (40.0)	4 (16.0)	0.038
Disease control rate	40 (88.9)	13 (52.0)	0.001

HPL, hepatic artery infusion chemotherapy combined with programmed cell death protein-1 inhibitors plus lenvatinb; PL, programmed cell death protein-1 inhibitors plus lenvatinib.

a Overall response included assessment of the change in tumor burden inside and outside the liver.

b fimmu.2021.619776Intrahepatic response only included assessment of the change in tumor burden inside the liver.

c Treatment response was assessed in evaluable patients.

### Safety Analysis

All AEs were evaluated as mild and manageable, and no toxicity-associated deaths occurred in the follow-up. More patients in the HPL group experienced grade 1–2 neutropenia and increased alanine aminotransferase. Only one patient experienced Grade 3 pain in the PL group. The details of the events were summarized in **Table 3**.

**Table 3:** Treatment-related adverse events.

Adverse events	Any grade			Grade 3/4		
	HPL (n=45)	PL (n=25)	P value	HPL (n=45)	PL (n=25)	P value
**Treatment-related AEs, n (%)**				0 (0)	1 (4.0)	0.357
Rash	3 (6.7)	2 (8.0)	1.000	0 (0)	0 (0)	1.000
Pruritus	3 (6.7)	2 (8.0)	1.000	0 (0)	0 (0)	1.000
Pain	7 (15.6)	7 (28.0)	0.212	0 (0)	1 (4.0)	0.357
Fever	12 (26.7)	5 (20.0)	0.500	0 (0)	0 (0)	1.000
Diarrhea	5 (11.1)	3 (12.0)	1.000	0 (0)	0 (0)	1.000
Fatigue	8 (17.8)	5 (20.0)	1.000	0 (0)	0 (0)	1.000
Nausea	6 (13.3)	1 (4.0)	0.408	0 (0)	0 (0)	1.000
Decreased appetite	8 (17.8)	2 (8.0)	0.314	0 (0)	0 (0)	1.000
Cough	4 (8.9)	3 (12.0)	0.694	0 (0)	0 (0)	1.000
Edema peripheral	2 (4.4)	1 (4.0)	1.000	0 (0)	0 (0)	1.000
Hypothyroidism	1 (2.2)	1 (4.0)	1.000	0 (0)	0 (0)	1.000
Hyperthyroidism	0 (0)	0 (0)	1.000	0 (0)	0 (0)	1.000
**Laboratory-related AEs, n (%)**				4 (8.9)	3 (12.0)	0.694
White blood cell count decreased	1 (2.2)	0 (0)	1.000	0 (0)	0 (0)	1.000
Hemoglobin decreased	2 (4.4)	0 (0)	0.534	0 (0)	0 (0)	1.000
Platelet count decreased	5 (11.1)	1 (4.0)	0.410	1 (2.2)	0 (0)	1.000
Neutropenia	7 (15.6)	0 (0)	0.045	0 (0)	0 (0)	1.000
Alanine aminotransferase increased	20 (44.5)	4 (16.0)	0.016	3 (6.7)	1 (4.0)	1.000
Aspertate aminotransferase increased	15 (33.3)	3 (12.0)	0.050	2 (4.4)	1 (4.0)	1.000
Total bilirubin increased	5 (11.1)	5 (20.0)	0.477	0 (0)	2 (8.0)	0.124
Albumin decreased	4 (8.9)	1 (4.0)	0.648	0 (0)	0 (0)	1.000
Creatinine increased	1 (2.2)	0 (0)	1.000	0 (0)	0 (0)	1.000

AEs, adverse events; HPL, hepatic artery infusion chemotherapy combined with programmed cell death protein-1 inhibitors plus lenvatinib; PL, programmed cell death protein-1 inhibitors plus lenvatinib.

### Prognostic Factor Analysis

The prognostic factors for survival are shown in **Table 4**. The comparison of PL to HPL was identified as an independent risk factor for both OS (HR = 3.180; 95% CI 1.608–6.290; p = 0.001) and PFS (HR = 2.702; 95% CI 1.440–5.070; p = 0.002). In addition, multivariate analyses identified that CP B and multiple tumors were risk factors for OS and that AFP ≥ 400 ng/ml was a risk factor for PFS.

**Table 4:** Univariate and multivariate analysis of risk factors for overall survival and progression-free survival.

Variables	Overall survival						Progression-free survival					
	Univariate analysis			Multivariate analysis			Univariate analysis			Multivariate analysis		
	HR	95% CI	*P*	HR	95% CI	*P* value	HR	95% CI	*P*	HR	95% CI	*P*
Age (y), (</≥50)	1.039	0.539–2.003	0.908				0.754	0.423–1.343	0.338			
Gender, (female/male)	0.413	0.413–2.007	0.816				1.509	0.702–3.240	0.292			
Hepatitis B, (no/yes)	1.194	0.516–2.762	0.679				1.438	0.669–3.089	0.352			
AFP (ng/ml), (</≥400)	1.788	0.896–3.569	0.099				2.096	1.144–3.840	0.017	2.896	1.507–5.568	0.001
PIVKA-II, (mAU/ml), (</≥400)	1.353	0.591–3.097	0.474				1.481	0.714–3.071	0.291			
Liver cirrhosis (no/yes)	1.225	0.476–3.153	0.674				1.188	0.531–2.660	0.674			
Child-Pugh (A/B)	4.309	1.501–12.373	0.007	3.709	1.239–11.099	0.019	2.109	0.754–5.897	0.155			
BCLC (B/C)	1.163	0.475–2.848	0.741				1.537	0.638–3.701	0.337			
Largest tumor size (cm),(<10/≥10)	1.670	0.837–3.333	0.146				1.531	0.845–2.773	0.160			
Tumor number (1/>1)	3.127	1.097–8.915	0.033	3.193	1.093-9.327	0.034	1.938	0.865–4.346	0.108			
Tumor distribution (uni-/bi-lobar)	1.337	1.029–1.737	0.030				1.322	1.062–1.647	0.013			
Tumor thrombus												
Absent												
Branch of portal vein	0.465	0.194–1.114	0.086				1.054	0.487–2.234	0.891			
Main portal vein	0.933	0.440–1.979	0.857				1.182	0.570–2.451	0.653			
Extrahepatic metastasis (no/yes)	1.569	0.819–3.003	0.174				1.033	0.578–1.846	0.912			
Treatment (HPL/PL)	2.770	1.437–5.340	0.002	3.180	1.608–6.290	0.001	1.865	1.044–3.330	0.035	2.702	1.440–5.070	0.002

AFP, alpha fetoprotein; PIVKA-II, protein induced by vitamin K absence or antagonist-II; BCLC, Barcelona Clínic Liver Cancer; PD-1, programmed cell death protein-1; HPL, hepatic artery infusion chemotherapy combined with PD-1 inhibitors plus lemvatinb; PL, PD-1 inhibitors plus lenva; HR, hazard rate; CI, confidence interval.

## Discussion

Treatment strategies for advanced HCC have progressed with the emergence of new tyrosine kinase inhibitors (TKIs) and immune-targeted therapy. Lenvatinib plus pembrolizumab has recently become a potent systemic combination therapy for unresectable HCC (6). In clinical practice, locoregional-systemic combinations are widely applied due to the overall control of tumor conditions (13). The result of a randomized clinical trial conducted by Ming Shi et al. demonstrated that a combination of sorafenib plus HAIC using FOLFOX agents extends overall survival by 87.5% or 6.24 months compared to sorafenib alone in HCC patients with portal vein invasion (9). Thus, HAIC may play a role in PL treatment. However, no research has reported the efficacy of HPL *versus* PL. Our retrospective study demonstrated that in advanced HCC, HPL results in a significantly better survival benefit than PL.

The efficacy benefit observed in the present study may be attributed to the synergistic antitumor effect of PD-1 inhibitors, lenvatinib, and FOLFOX agents. Oxaliplatin induces immunogenic cell death in HCC cells and synergizes with PD-1 targeted immunotherapy (14). Lenvatinib inhibits multiple receptor tyrosine kinases (RTKs) targeting VEGFR1-3, FGFR1-4, PDGFR α, RET, and KIT (15). On the one hand, inhibition of VEGFR and FGFR elicits antitumor immunity and enhances PD-1 checkpoint blockade in HCC (16). On the other hand, antiangiogenesis normalizes tumor vessels and breaks the hypoxic microenvironment of tumors, thereby attenuating the activity of chemoresistance (17–19).

In this study, the median OS and PFS were 8.6 months and 5.4 months in the PL group, respectively, which were better than those observed in a sorafenib monotherapy trial in the Asia-Pacific region (20). However, the survival outcomes were far worse than those in the Keynote-524 trial (6). Worldwide trials of PD-1 inhibitors or lenvatinib monotherapy in advanced HCC have shown a better OS over 1 year (21, 22). Compared to these studies, the patients included in our study were relatively more late-staged with the majority of the patients in the PL group classified with BCLC stage C (88%), major (48%) or branch (24%) of portal vein tumor thrombus, extrahepatic metastases (52%) and tumor burden over 10 cm (60%). In contrast, the median OS and PFS were significantly better in the homogeneous patients in the HPL group, implying efficacy for the HPL therapy.

The treatment response showed significantly higher ORR and DCR in the HPL group compared to the PL group. Of note, eight patients were unable to assess tumor response in the PL group. One unavoidable reason was that patients treated with systemic medications were not hospitalized, causing the relatively high rate of missed imaging examinations during the treatment, which affected the accurate assessment of tumor response rates. Thus, this variable needs to be further controlled in prospective studies.

In the subgroup analysis, significant differences were not reached in certain subgroups with small proportional cohorts due to limitations in the number of cases. In general, HPL *versus* PL provided a survival advantage in patients with multiple tumors and tumor diameters greater than 10 cm, but HPL was less effective in patients with main portal vein tumor thrombus and extrahepatic metastases. These findings suggested that HAIC, as a locoregional approach, has a great ability to control intrahepatic lesions but that it may fail to manage extrahepatic metastases. Univariate and multivariate Cox regression analyses showed different factors associated with OS and PFS. This may be partly due to the incongruity between progression and survival in the combination therapy of advanced HCC. Patients with the progressive disease could receive more treatment and get inconsistent survival benefit. Of note, HPL was an independent prognostic indicator for both OS and PFS, which confirmed the positive efficacy of HAIC in the combination therapy of PL.

The present study had some limitations. First, the study was a retrospective study in a single center, resulting in inevitable selection bias. Second, the PD-1 inhibitors were varied, which influenced the uniformity of the treatment procedure. Third, the number of cases was relatively small. Findings from this study should be further expanded to a multicenter study to obtain higher-level medical evidence.

Based on our results, HPL is associated with a significantly better treatment response and survival benefits compared to PL. Thus, HPL may be a potential new treatment option for advanced HCC.

## Data Availability Statement

The original contributions presented in the study are included in the article/**Supplementary Material**. Further inquiries can be directed to the corresponding authors.

## Ethics Statement

This study was conducted according to the ethical guidelines of the 1975 Declaration of Helsinki. The analysis of the patient data was reviewed and approved by the Institutional Review Board and Human Ethics Committee at the Sun Yat-sen University Cancer Center (SYSUCC; Guangzhou, China)****.

## Author Contributions

R-PG, WW, and S-HL designed the study. JM and Y-HT collected the data. JM, Y-HT, MS, WW, S-HL, and R-PG analyzed and interpreted the data. LZ performed the radiological evaluation. JM, Y-HT, S-HL, and R-PG prepared the final draft. All authors contributed to the article and approved the submitted version.

## Conflict of Interest

The authors declare that the research was conducted in the absence of any commercial or financial relationships that could be construed as a potential conflict of interest.****
