# Supplementary material for: Hepatic Arterial Infusion Chemotherapy Combined With PD-1 Inhibitors Plus Lenvatinib Versus PD-1 Inhibitors Plus Lenvatinib for Advanced Hepatocellular Carcinoma
Source: Front Oncol. 2021 Feb 25;11:618206. doi: 10.3389/fonc.2021.618206 (PMC7947809; doi:10.3389/fonc.2021.618206)
Supplement: Supplementary file 1 [file Table_1.docx]

Supplement Tables

Table S1. Category and dosage of PD-1 inhibitors used in the two groups.

| **Category** | **Dose**  **(mg)** | **HPL**  **(n=45)** | **PL**  **(n=25)** |
| --- | --- | --- | --- |
| Nivolumab | 100 | 2 (4.4) * | 5 (20.0) |
| (Bristol-Myers Squibb) |  |  |  |
| Keytruda | 200 | 4 (8.9) | 7 (28.0) |
| (Carlow, Merck Sharp & Dohme Corp) |  |  |  |
| Toripalimab | 240 | 40 (88.9) | 13 (52.0) |
| (Suzhou, hezhong pharmaceutical Co.Ltd) |  |  |  |
| Sintilimab | 200 | 1 (2.2) | 6 (24.0) |
| (Suzhou, xinda pharmaceutical Co.Ltd)) |  |  |  |

*No (%)

HPL: hepatic artery infusion chemotherapy combined with PD-1 inhibitors plus lemvatinb; PL: PD-1 inhibitors plus lenvatinib
